# Feather and skin development of ostriches *Struthio camelus*

**DOI:** 10.4102/jsava.v89i0.1556

**Published:** 2018-12-05

**Authors:** Tertius S. Brand, Werné J. Kritzinger, Daniel A. van der Merwe, Anieka Muller, Louw C. Hoffman, Gert J. Niemann

**Affiliations:** 1 Directorate for Animal Sciences, Western Cape Department of Agriculture, South Africa; 2 Department of Animal Science, Stellenbosch University, South Africa

## Abstract

Information on feather and skin growth is important for the development of mathematical optimisation nutritional models for ostriches. Ostriches (*n* = 65) were subjected to a four-stage formulated growth diet programme (pre-starter, starter, grower and finisher), with declining protein and energy content. Nine birds were weighed, stunned, exsanguinated, defeathered, skinned and eviscerated at 1, 54, 84, 104, 115, 132 and 287 days of age. Feathers from four pre-selected locations on the body were harvested and weighed. The wet skin weight, wet unstretched skin size and wet unstretched crown size were measured at each slaughter stage. The live weight, feather and skin yields of the birds increased with age at slaughter, as did feather shaft diameter. Prediction models were developed to estimate the yield of the skin in terms of live weight and of empty body protein weight to aid in diet formulation. The allometry of feather growth was determined from total feather weight, as the maturation rates of the feathers differ from that of the ostrich body. Results from this study will aid in setting up a mathematical optimisation nutritional model for ostriches.

## Introduction

A model can be defined as the simulation of a system that enables predictions that are as close to reality as possible. A description of reality is needed to set up an accurate prediction model (Emmans & Fisher 1986).

Emmans (1989, 1995) and Ferguson (2006) stated that a bird will attempt to grow at its absolute genetic potential. The achievement of this goal is influenced by environmental conditions, state of the animal, nutritional and physical factors such as gut capacity and intake regulation (Ferguson 2006). Growth of the empty body (without gut fill) can be seen as the weight accumulation of protein, ash, water and lipid, while biological tissue growth follows a sigmoidal pattern (Huxley 1932; McDonald et al. 2002). These tissues and components are allometrically related. Relating all components to a standard factor like the featherless empty body protein weight (EBPW) will provide a way to compare and predict body component growth (Emmans 1989; Ferguson 2006).

Feather growth needs to be separated from body growth, as feather protein differs from body protein in amino acid composition. Emmans (1989) reported that feather protein contains high levels of cystine (70 g/kg protein) and low levels of lysine (18 g/kg protein), whereas body protein contains low levels of cystine (11 g/kg protein) and higher lysine levels (75 g/kg protein). In addition, the feathers mature at different rates from the body components (Emmans 1989; Emmans & Fisher 1986). Feather protein growth and body protein growth are thus not allometrically related. Consequently, the proportion of feather protein in the total body protein changes as the bird matures; therefore, predictions for feather protein growth cannot be made from body protein growth. The description of feather growth will help with cost-effective amino acid formulations in changing diets during the production cycle.

The aim of this study was to define the factors affecting income-generating products by setting up equations to model skin and feather development in growing ostriches.

## Materials and methods

Ostriches (*n* = 65) were reared under standard commercial practices, with nine randomly selected ostriches slaughtered at 1, 54, 85, 104, 115, 132 and 287 days of age. The birds were reared in 13 pens with *ad libitum* access to feed and water available throughout the trial period. The diets for the four growth sages, pre-starter (0–1 months), starter (1–3 months), grower (3–6 months) and finisher (6–9 months) were formulated according to set specifications (Gous & Brand 2008) (Table 1). The feeds were sampled and analysed (AOAC 2002) for protein, amino acids, fat, crude fibre, neutral detergent fibre (NDF), acid detergent fibre (ADF) and ash (Table 1). The trial was terminated when the last group of ostriches was slaughtered at 287 days of age.

**TABLE 1:** The feed ingredients and nutrient composition of the formulated feeds fed to the ostriches.

Variable	Feeding stage			
	Pre-starter	Starter	Grower	Finisher
**Ingredients (g/kg)**				
Maize meal	500.00	200.00	-	-
Barley grain	-	400.00	400.00	200.00
Wheat bran	50.00	-	-	-
Lucerne meal	98.00	143.00	398.00	700.00
Molasses powder	-	-	25.00	25.00
Sunflower oil-cake meal	-	-	100.00	-
Soya bean oil-cake meal	96.00	80.00	50.00	50.00
Full-fat soya meal	71.00	50.00	-	-
Fish meal	150.00	75.00	-	-
Plant oil	10.00	10.00	-	-
Synthetic lysine	-	0.90	0.60	-
Synthetic methionine	0.70	0.50	1.00	0.90
Mono-calcium phosphate	-	14.00	1.00	6.00
Limestone	15.00	18.00	15.00	7.00
Vitamin and mineral mix	5.00	5.00	5.00	5.00
Salt	4.00	4.00	4.00	4.00
Total	1000.00	1000.00	1000.00	1000.00
**Nutrients**				
Dry matter (%)	90.90	91.80	91.10	90.70
ME_ostrich_ (MJ/kg)	14.42	13.50	11.26	10.06
Crude protein (g/kg)	248.00	223.00	181.00	155.00
Fat (g/kg)	60.00	45.00	39.00	23.00
Ash (g/kg)	90.00	99.00	71.00	84.00
Fibre (g/kg)	65.00	97.00	82.00	199.00
ADF (g/kg)	92.00	129.00	110.00	222.00
NDF (g/kg)	166.00	197.00	197.00	342.00
Lysine (g/kg)	13.00	10.00	8.00	5.00
Methionine (g/kg)	5.00	4.00	3.00	2.00
Cysteine (g/kg)	2.00	2.00	2.00	1.00
Threonine (g/kg)	12.00	11.00	9.00	7.00
Arginine (g/kg)	12.00	10.00	9.00	6.00

Note: Pre-starter, 0–1 months; Starter, 1–3 months; Grower, 3–6 months; Finisher, 6–9 months.

ADF, acid detergent fibre; NDF, neutral detergent fibre; ME, Metabolizable Energy.

At each slaughter age, each bird was weighed, stunned, exsanguinated, defeathered and eviscerated. The feathers from four regions were collected separately and dried in a drying oven at 80°C for 48 h. These four regions included the wing feathers (white plumes, the first row of big plumes found on edge of the wings), byocks (the pied feathers at each end of the row of white plumes), tail feathers (80–100 larger feathers at the caudal tail region) and the drab floss (short body floss, feathers that are primarily found on the line above the pelvic joint as well as in the centre of the dorsal surface of the wing just before the long hard body feathers). This study was based on the total feather weight of the birds at each slaughter age and no adjustments were made for feathers lost during general animal husbandry practices.

After drying, feathers were weighed separately according to the body region. The shafts of ten randomly selected wing feathers from each bird were measured at the base (point of skin entry) using digital callipers. At slaughter, after removal of the feathers, the skin was flayed, weighed and the surface area determined by spreading the wet skin over a linen cloth and tracing the outlines. The traced version was cut out and the surface area determined by means of computerised video image analysis.

The gastrointestinal tract of each bird was rinsed clean with water and weighed. The heart, liver and 13 muscles were removed and individually weighed. The neck (separated at the last cervical vertebra), leg (femur, patella and tibiotarsus), wingtip (metacarpus and digiti manus) and rib cage bones were weighed. After weighing, all of the components for each bird, along with the blood feathers, were frozen in separate plastic bags. The body as a whole was grounded through an industrial grinder and mixed thoroughly. A randomised sample of the ground mixture was collected (~150 g) to perform proximate analysis by the Association of Official Analytical Chemists methods (AOAC 2002) so as to determine the EBPW.

As gut fill varies between individuals and can account for 8% – 15% of live weight (Swart, Mackie & Hayes 1993), the empty body weight is preferred as a measure of the size of the ostrich. It is recommended that feather protein and body protein should be analysed separately because of differences in amino acid composition (Emmans 1989), which was not carried out in this study. To correct for this, ostrich feathers from a separate investigation (unpublished data) were analysed and the proportional protein contribution of feathers was deducted from the previous protein analysis in order to give the featherless EBPW, which was used in the statistical analysis.

The statistical analyses were performed using the common slope procedure of Statistical Analysis Systems software version 9.1 (SAS Institute Inc., Cary, NC, United States) (SAS 2000). An adapted form of the Gompertz growth curve of the form *y* = a*(exp(-exp(-b*(age-*c*))) (Emmans 1989) was fitted to the skin size data in order to confirm the sigmoidal growth pattern. Linear data were obtained by transformation into the natural logarithmic form (Huxley 1932; Lawrie 1998; McDonald et al. 2002). The natural logarithm of the skin size (dm^2^) and weight (kg) were regressed against the natural logarithm of EBPW. The weights from each feather region (kg) were regressed against the total feather weight (kg).

### Ethical considerations

This study (project number R10/13) was approved by the Departmental Ethical Committee for Research on Animals (DECRA).

## Results

The shaft diameter of the wing feathers and the weights of the different feathering regions along with the accompanying standard deviations are presented in Table 2. Results show that there was a general increase in the shaft diameter and various feather weights with age. The shaft diameter seemed to increase up until 132 days of age (5.95 mm ± 0.84 mm), after which it remained relatively constant. Of the four feather regions sampled, wing feathers contributed the greatest proportion in weight towards the total feather weight, with the heaviest weights being achieved at 132 and 287 days of age (~245 g). It was observed that the wing feather weights were similar between days 85 and 104 of age (35.1 g ± 19.1 g and 36.6 g ± 23.5 g, respectively), whereas byocks and tail feather weights were seen to have increased drastically at these slaughter ages.

**TABLE 2:** The shaft diameter of the wing feathers and the feather weight (dry matter basis) at each region, and the accompanying standard deviations for every slaughter age.

Age (days)	Average wing shaft diameter (mm)	Feather weight (g)				
		Drab floss†	Byocks‡	Tail§	Wing¶	Total feathers
1	0.14 ± 0.03	0.03 ± 0.01	0.04 ± 0.01	0.22 ± 0.08	1.2 ± 0.5	12.90 ± 3.6
54	1.79 ± 0.38	0.20 ± 0.10	2.80 ± 2.10	1.20 ± 1.10	7.9 ± 6.9	38.80 ± 29.2
85	3.39 ± 0.49	0.60 ± 0.30	7.50 ± 5.40	4.80 ± 2.70	35.1 ± 19.1	100.40 ± 39.5
104	2.85 ± 0.68	1.10 ± 0.60	22.50 ± 16.40	19.10 ± 15.90	36.6 ± 23.5	154.15 ± 65.6
115	5.47 ± 0.55	3.20 ± 1.70	48.00 ± 10.90	19.20 ± 5.90	112.2 ± 29.6	336.00 ± 82.3
132	5.95 ± 0.84	27.10 ± 4.70	85.30 ± 14.70	39.70 ± 18.40	246.9 ± 75.7	653.00 ± 168.4
287	4.91 ± 0.51	37.20 ± 17.80	140.00 ± 29.40	109.60 ± 40.40	245.6 ± 54.7	836.60 ± 128.2

† , Drab floss (short body floss) – primarily found on the line above the buttocks joint, as well as in the centre of the dorsal surface of the wing just before the long hard body feathers;

‡ , Byocks – pied feathers at each end of the row of white feathers.

§ , Tail feathers – 80–100 larger feathers on the tail.

¶ , Wing feathers (white plumes) – first row of big plumes at the wing edge.

The allometric relationship of the growth of the feathering regions, given by the natural logarithms, with the natural logarithm of the total feather weight was described using linear regressions (Table 3). The coefficients of determination (*R*^2^) for each of these regressions were high (> 0.90), showing that most of the variations for the allometric coefficients for the growth of these four feather regions were accounted for by these models.

**TABLE 3:** Allometric coefficients relating the natural logarithms of the weighed feathers to the natural logarithms of the total feather weight.

Component	Constant term	Regression coefficient	*R*^2^
Drab floss	−7.8518 ± 0.3268	1.6550 ± 0.0595	0.94
Byocks	−6.6623 ± 0.4363	1.7621 ± 0.0794	0.91
Tail	−4.9332 ± 0.2299	1.3777 ± 0.0419	0.96
Wing	−2.9282 ± 0.1239	1.2877 ± 0.0226	0.99

Table 4 shows the average live weights and skin sizes of the ostriches at the respective slaughter ages. The live weights of the ostriches increased with age. Wet skin size, wet crown size and wet skin weight are presented in Table 4. The average wet skin size in this study at day 287 or about 10 months, when ostriches are typically slaughtered, was 96.70 dm^2^. The wet skin size from this study can be converted to the dry crust size by the function:

*y* = 0.791587*x* + 52.31, *R*^2^ = 0.64. (Brand 2008).

**TABLE 4:** The wet skin data and the accompanying standard deviations at each slaughter age and slaughter weight.

Slaughter age (days)	Live weight (kg)	Wet skin weight (kg)	Wet unstretched skin size (dm^2^)	Wet unstretched crown size (dm^2^)
1	0.86 ± 0.07	0.08 ± 0.02	5.50 ± 0.53	-
54	6.28 ± 2.80	0.35 ± 0.10	19.88 ± 5.89	6.38 ± 1.70
85	9.39 ± 3.80	0.46 ± 0.20	25.00 ± 9.97	9.00 ± 3.80
104	13.20 ± 4.40	0.57 ± 0.20	27.25 ± 5.56	8.75 ± 1.90
115	21.60 ± 2.80	0.73 ± 0.10	35.00 ± 3.97	12.20 ± 1.90
132	35.40 ± 7.90	1.88 ± 0.60	61.44 ± 11.10	21.38 ± 4.60
287	73.90 ± 9.30	3.91 ± 0.60	96.70 ± 13.27	32.30 ± 4.16

kg, kilograms; dm^2^, decimeters^2^.

**FIGURE 1:**
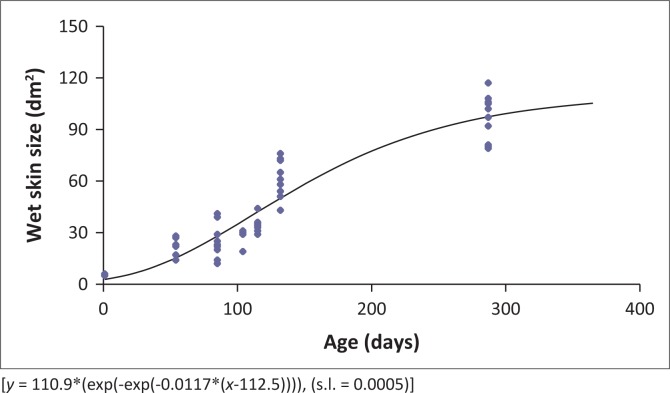
A Gompertz model fitted to the wet skin size increase against age for ostriches.

This converts the average wet skin size at 10 months of age to a crust size of 128.9 dm^2^ ± 10.5 dm^2^. A Gompertz growth model was fitted to the data of the wet skin sizes recorded in this study (Figure 1). The wet skin size increased in a sigmoidal pattern, as described by the Gompertz function. This function can be used to predict the wet skin size of an ostrich at a particular age, which, in turn, can be converted to dry crust size using the above equation. This illustrates a maximum wet skin size of 110.9 dm^3^ (predicted crust size of 140.1 dm^3^) and the age of maximum growth of the skin at 112.5 days of age.

In order to further predict skin yield, the natural logarithms of the wet skin size and skin weight (kg) were related to the natural logarithms of the EBPW. Linear regressions of the form *y* = *ax* + *b* (where *y* represents the natural logarithms of either skin size or weight and *x* represents the natural logarithm of EPBW) were developed with high *R*^2^ coefficients of determination (0.96 and 0.92, respectively).

## Discussion

The ostrich industry and the importance of the three products derived from ostrich production (meat, leather and feathers) are constantly changing in economic value. To maintain profitability of an ostrich production enterprise, it is of utmost importance to be able to predict the growth and characteristics of the feathers and skins of growing ostriches. The feather regions sampled in this study consist of the feather classes that have the greatest economic value in terms of feather production. Thus, it is essential that the yields of these feathers can be predicted from the total amount of feathers harvested (Table 3). The feather growth was thus defined in terms of the total feather weight rather than from live weight or EBPW. Feather growth is also defined separately from EBPW as the components differ both in growth rate (Emmans 1989; Emmans & Fisher 1986) and in their amino acid composition (Emmans 1989).

Feathers mature at an earlier rate than the body (Emmans & Fisher 1986). While the proportions of the other feathering regions continued to increase throughout the study period, the weight of wing feathers collected after 132 days of age did not increase. This possibly indicates that the wing feathers may have reached a state of maturity at this point (Table 2), although further investigations on older birds as well as collecting more data between 132 and 287 days of age would be essential to attempt to confirm a plateauing of feather weight. The shaft diameters of the wing feathers also seemed to plateau from about 132 days of age.

The skin of the ostrich currently has the greatest economic value of the three products that can be derived from the ostrich. The skin is subjected to a tanning process to yield a high-quality leather crust (because of the nodules that develop on the skin from which the feathers grow) with high aesthetic appeal. The value of ostrich leather is determined primarily by the size of the crust and its appearance, which is judged by the level of damage found on the skin and the size of the feather nodules. As expected, as the ostrich ages and increases in live weight, the yield of the skin (in terms of surface area and weight) increases. The results from this investigation (Table 4) correspond with previous findings (Mellett 1992), where an acceptable skin size of between 120 dm^2^ and 124.29 dm^2^ was reported to be obtained at the age of 10 months.

As the wet skin size increases with age in a nonlinear fashion, the Gompertz function could describe the increase in skin surface area of the growing bird (Figure 1). This is in contrast to the findings of Cloete et al. (2004) and Van Schalkwyk et al. (2002), who reported that ostrich raw skin yield increases linearly with age. This study indicates a mature skin size of 140.1 dm^3^ and maximum growth of the skin at 112.5 days of age. When expressing these yields in relation to the EBPW, the given allometric equations in Table 5 will render a portrayal of growth that will aid in predicting the yields that can be expected and can be used to model the nutrient requirements of the birds at different stages in the growth cycle. The size of the leather crust can also be deduced from the weight skin weight using the model proposed by T.S. Brand (unpublished results, 2008).

**TABLE 5:** Allometric equations relating the natural logarithms of the wet skin size and weight to the natural logarithms of the empty body protein weight (kg).

Component	Constant term	Regression coefficient	*R*^2^
Wet skin size (dm^2^)	2.8186 ± 0.0291	0.5885 ± 0.0162	0.96
Wet skin weight (kg)	−1.1960 ± 0.0558	0.7938 ± 0.0311	0.92

kg, kilograms; dm^2^, decimeters^2^.

Table 6 presents correlation coefficients (*r*) between a range of explanatory variables related to a model to predict feather and skin development in ostriches. The data confirm the close relationship between the development of these variables.

**TABLE 6:** Correlation coefficients (*r*) between a range of explanatory variables related to a model to predict feather and skin development in ostriches.

Variable	Live weight (kg)	Age (days)	Wing shaft diameter (mm)	Total feathers (g)	Skin area (dm^2^)	Skin weight (kg)	EBPW (kg)
Live weight (kg)	1.00	0.96*	0.63*	0.94*	0.98*	0.98*	1.00*
Age (days)	-	1.00	0.65*	0.89*	0.94*	0.93*	0.96*
Wing shaft diameter (mm)	-	-	1.00	0.76*	0.69*	0.56*	0.67*
Total feathers (g)	-	-	-	1.00	0.96*	0.92*	0.94*
Skin area (dm^2^)	-	-	-	-	1.00	0.97*	0.97*
Skin weight (kg)	-	-	-	-	-	1.00	0.97*
EBPW (kg)	-	-	-	-	-	-	1.00

* , All correlations are significant at *p* < 0.05.

EBPW, empty body protein weight; kg, kilograms; mm, millimetres; dm^2^, decimeters^2^.

The equations developed in this study for slaughter ostrich systems can also be used to predict the yields of feathers and skins that can be obtained from an ostrich slaughtered within 10 months of age. The models relating to the EBPW can also be used to aid nutritionists in formulating diets to meet the requirements for skin growth to yield an optimal leather crust.

## Conclusion

The allometric equations provided in this study offer the ability to predict feather and skin yield in ostriches through most of the growth cycle, in order to enhance profitability of the production system. Leather yield is affected by skin size, which currently reaches acceptable marketing size at approximately 10 months of age. This is, however, related to variation in market requirements, especially in terms of nodule size. This, along with the cyclical nature of the leather, meat and feather prices, together with the careful consideration of raw feed ingredient prices, could be used to optimise the economical facets of ostrich production. More research is needed in the field of nodule growth and shape prediction, and a model that predicts nodule size in the live bird up to 16 months of age is required. Results in this study, along with future research, could form the basis for constructing a simulation model that accurately predicts changing nutrient requirements in the slaughter off ostriches at different ages.
